# The Effects of Organically Modified Lithium Magnesium Silicate on the Rheological Properties of Water-Based Drilling Fluids

**DOI:** 10.3390/ma17071564

**Published:** 2024-03-29

**Authors:** Taotao Luo, Jun Li, Jiangen Xu, Jun Wang, Lianxi Zhang, Zeya Yu

**Affiliations:** Institute of Petroleum and Natural Gas Engineering, Chongqing University of Science and Technology, Chongqing 401331, China; 2017039@cqust.edu.cn (T.L.);

**Keywords:** composites, weak gel, viscosity enhancer, resistant to high temperature and salt, drilling fluids

## Abstract

To address the problem of insufficient temperature and salt resistance of existing polymer viscosity enhancers, we designed an organic–inorganic hybrid composite as a viscosity enhancer for water-based drilling fluids, named LAZ, and it was prepared by combining a water-soluble monomer and lithium magnesium silicate (LMS) using an intercalation polymerization method. The composite LAZ was characterized using Fourier transform infrared spectroscopy, transformed target X-ray diffractometry, scanning electron microscopy, and thermogravimetric analysis. The rheological properties of the composite LAZ were evaluated. The composite LAZ was used as a water-based drilling fluid viscosity enhancer, and the temperature and salt resistance of the drilling fluid were evaluated. The results showed that the composite LAZ presented a complex reticulation structure in an aqueous solution. This reticulation structure intertwined with each other exhibited viscosity-enhancing properties, which can enhance the suspension properties of water-based drilling fluids. The aqueous solution of the composite LAZ has shear dilution properties. As shear rate increases, shear stress becomes larger. The yield stress value of the aqueous solution increases as the composite LAZ’s concentration increases. The aqueous solution of the composite LAZ exhibits strong elastic characteristics with weak gel properties. The addition of the composite LAZ to 4% sodium bentonite-based slurry significantly increased the apparent viscosity and dynamic shear of the drilling fluid. The drilling fluids containing the composite LAZ had good temperature resistance at 150 °C and below. The rheological properties of brine drilling fluids containing the composite LAZ changed slightly before and after high-temperature aging at 150 °C.

## 1. Introduction

Conventional water-based drilling fluids have been widely used in drilling projects. Water-based drilling fluids are generally dispersed systems consisting of clay and water, which, in addition to clay and water, usually contain soluble salts, various types of functional agents, and weighting agents [1,2]. During drilling fluid circulation, good drilling fluid rheology can quickly and efficiently remove rock cuttings deposited at the bottom of the well and bring them to the surface, increasing mechanical drilling speed. When the pump is stopped and resting, good drilling fluid rheology can make the drilling fluid effectively suspend solid-phase particles and prevent the settling of weighting materials and rock cuttings. During the circulation process, good rheological properties can reduce the erosion of the drilling fluid on the borehole wall, preventing the wall from collapsing due to fluid erosion, and have a stabilizing role [3,4,5].

Adding polymer materials to the drilling fluid and dissolving and dispersing it in the aqueous phase can form a reversible spatial reticulated structure in the drilling fluid. This structure gives the drilling fluid the appropriate viscosity and thixotropic and other rheo-logical properties to ensure the high efficiency of drilling wells and prevent accidents caused by the high pump pressure when turning on the pump [6,7,8]. At the same time, these polymer materials can also be retained or adsorbed in the pore space of the formation, reducing the filtration loss of the drilling fluid and improving the blocking performance. In this way, they reduce the damage of the drilling fluid to the oil formation.

Navarrete [9] explored how the rheological behavior of xanthan-based fluids can be used to control fluid loss. Cores of three different permeability ranges representing ~100 mD, ~600 mD, and ~1 D were used in the tests. Linear and radial flow tests were performed on rocks. Sean Robert Smith [10] evaluated the apparent viscosity (AV), plastic viscosity (PV), yield point (YP), gel strength after 10 s and 10 min (Gel 10 s and Gel 10 min), and filtration properties of water-based fluids (WBFs) with added aluminum oxide at both low- and high-temperature conditions as standard practice of drilling fluids characterizations. Zisis Vryzas [11] shed some additional light on the mechanisms of the bentonite interparticle interaction in high-temperature environments by combining macroscopic measurements (rheological measurements) with microscopic measurements (e.g., X-ray diffractometry (XRD), scanning electron microscopy (SEM), and transmission electron microscope (TEM), which can aid our understanding of the interactions of the particles in the microcosmos of such dispersions.

Westland et al. [12] developed novel entangled reticulated cellulose (RBC) through biotechnology. This cellulose belongs to a special category of cellulose that is different from the traditional cellulose, which originates from bacteria. After adding RBC into the water-based drilling fluid, the drilling fluid showed excellent rheological properties, which can improve the transportation performance of the weighting agent and rock cuttings under static and dynamic conditions, has a certain temperature and salt resistance, and has better anti-shear stability. Cobianco et al. [13] prepared a new polymer treatment agent that can be added to drilling fluids, which showed special rheological properties. Other functional treatment agents were screened through indoor experiments to establish new clay-free drilling fluids with weak gel properties. The special rheology of this drilling fluid plays a very important role in solving the problems of dynamic rock cuttings carrying, static rock cuttings suspending, and improving the mechanical drilling speed during the drilling process.

Dobson et al. [14] created a new weak gel-type drilling fluid system using biopolymer species as a viscosity builder. This weak gel drilling fluid showed good results for low-permeability reservoirs. The drilling fluid can quickly form a dense mud cake at the well wall formation during drilling through the synergistic effect of biopolymers, highly cross-linked starch, and micro-structured cellulose. This method can effectively prevent the invasion of solid particles to achieve the effect of reservoir protection. Xianbin Huang [15] synthesized a terpolymer (AAD) through a radical polymerization of acrylamide, 2-acrylamido-2-methylpropane sulfonic acid, and diallyldimethylammnium chloride monomers and studied the thermogravimetric analysis (TGA) of AAD/laponite nanocomposites and viscosity changes as a function of the corresponding solutions’ temperature and evaluated the drilling fluids’ performances.

Mao et al. [16] synthesized a hydrophobic associative micro-nano polymer based on silica nanoparticles having a shell-core structure by inverse emulsion polymerization with 2-acrylamido-2-methyl-propanesulfonic acid (AMPS), maleic anhydride (MA), and styrene and silica nanoparticles. The plugging ability of the polymer to micro-pores and micro-fractures and the maintenance of wellbore stability were evaluated through simulated drilling fluid displacement core column experiments. The test results showed that the micro-nano polymer had excellent performance in terms of thermal stability, rheological property, filtration property, and lubricity. The micro-nano polymer could effectively seal the formation and improve its bearing capacity. Ponmani et al. [17] used special polymer agents as viscosity-increasing and lifting cuttings for drilling fluids to obtain unique rheological properties and form a clay-free drilling fluid with good performance and weak gel properties. The rheological properties of the drilling fluid play a critical role in solving the problems of rock cuttings carrying during the dynamic process, rock cuttings suspending during the static process, and improving the mechanical drilling speed during the drilling process.

The unique rheological properties of drilling fluids can be obtained by using polymers with special structures as viscosity enhancers for drilling fluids. Composite materials formed by combining inorganic materials and organic polymers are attractive for creating high-performance or high-functional polymeric materials. Of particular interest is the molecular-level combination of two different components that may lead to new composite materials, termed “organic–inorganic hybrid materials” [18].

Schmidt et al. [19] introduced the concept of organic–inorganic hybrid materials, where strong forces exist between the two phases, or where an interpenetrating network structure is formed. Organic materials have better flexibility, easy processing and molding, and good impact resistance but poor long-term stability. In contrast, inorganic materials have better temperature resistance, strength, long-term stability, long service life, and other advantages but are difficult to mold. If the inorganic and organic materials are compounded, composite polymer materials with excellent performance can be obtained. Jun Li et al. [20] studied a composite gel that was formed by the hydration of bentonite and polyacrylamide under the action of an organic crosslinking agent. The particle size and microstructure of the composite gel were studied, and the internal structure and mechanics of the composite hydrogel were analyzed using rheological methods. Hasmukh A et al. [21] designed and synthesized layered materials that have covalently linked organic functionalities. Two types of synthetic magnesium silicates (MSils) were prepared, bearing hexadecyl (MSil-C16) or phenyl groups (MSil-Ph) through a facile synthetic route. The formation of layered structures was evaluated by X-ray diffraction, and covalent bonding of organic moieties with nanometer thickness magnesium silicates was revealed by infrared spectroscopic analyses. The thermal stabilities of MSils were studied by thermogravimetric analysis. Rabia Ikram’s [22] study is the first to reports the application of welding waste and its derived graphene oxide (GO) as a fluid-loss additive in drilling fluids. In this research, GO was successfully synthesized from welding waste through chemical exfoliation. The examination was confirmed using XRD, FTIR, FESEM, and EDX analyses. Quande Wang [23] constructed a green and simple adsorption approach to prepare highly dispersed graphite using a cationic surfactant for graphite modification and evaluated the performance of the modified graphite as a lubricated additive in water-based drilling through a rheological study and viscosity coefficient measurement.

In recent years, with the development of deep oil and gas resources, the drilling fluids in the wells will be in a high temperature and salt environment for a long time. Therefore, the resistance to temperature and salt is particularly important. Organic polymer viscosity enhancers degrade at high temperatures and in salt, causing the viscosity of drilling fluids to decrease, resulting in poor suspension and rock-carrying capacity of drilling fluids. Therefore, to solve the problem of water-based drilling fluids in extreme conditions due to the failure of polymer viscosity enhancers, it is necessary to develop a new type of polymer materials as drilling fluid viscosity enhancers. In this study, an organic–inorganic composite material was formed by combining an intercalation-modified LMS with a water-soluble reactive monomer to improve temperature and salt resistance. The role of the composite material as a viscosity enhancer in drilling fluids was investigated as an agent for the rheological properties of drilling fluids.

## 2. Experimental Methodology

### 2.1. Chemicals and Characteristics

All the chemicals used were of analytical grade and used as received. Lithium magnesium silicate (LMS) and 2-Acrylamido-2-methylpropane sulfonic acid (AMPS) were purchased from Shandong Yousuo Chemical Technology Co., Ltd. (Linyi, China). Cetyltrimethylammonium bromide (CTAB) was obtained from Tianjin Damao Chemical Reagent Factory. (Tianjin, China). Acrylamide (AM) and acrylic acid (AA) were purchased from Chengdu Kelong Chemical Company (Chengdu, China).

LMS is one of the commonly used reagents for drilling fluid additives because of its good suspending and thixotropic properties, strong blocking ability, and non-toxicity. AM and AA are the main monomers for synthesizing water-soluble polymers. AMPS contains a hydration group, sulfonic acid group, and sulfonic acid group, and effectively solves problems such as salt resistance, high temperature resistance; it has been applied to polymer modification in various fields of oilfield chemicals. CTAB can enter the interlayer structure of layered silicate materials, increasing the interlayer distance.

### 2.2. Synthesis and Characterization

The synthesis process of the composite material was mainly divided into two steps: the first step was intercalation modification of LMS, and the second step was the polymerization reaction. Interpolation modification experimental steps included weighing 2 g of LMS and dispersing it in 50 mL of deionized water to obtain an inorganic gel solution. The inorganic gel solution was heated to 40 °C, continuously heated for 30 min, and the temperature was raised to 65 °C. Then, under the action of stirring, CTAB was slowly added to the inorganic gel solution; the reaction was performed for 2 h, followed by drying to obtain the intercalation-modified LMS.

**Figure 1:**
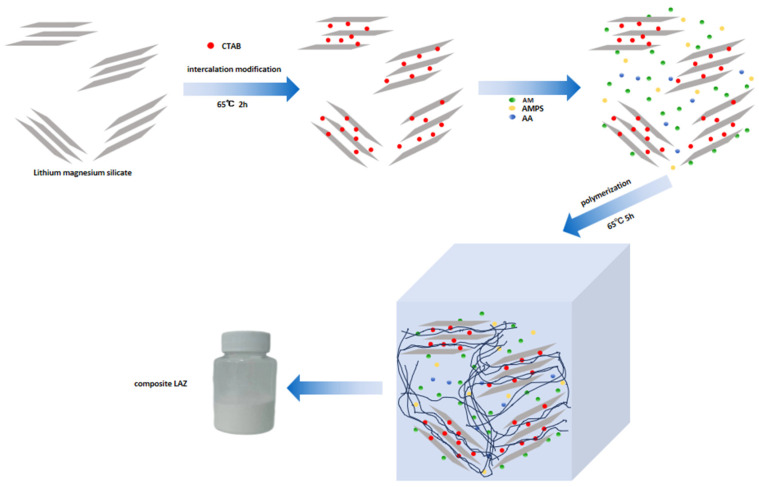
Preparation process of the composite LAZ.

The polymerization experimental steps included weighing the reaction monomers 7.5 g of AM, 1.25 g of AMPS, and 3.75 g of AA, and dispersing in 50 mL of deionized water and stirred for 15 min to make the reaction monomers dispersed and dissolved. Sodium hydroxide adjusted the pH of the reactive monomer solution to neutral. The aqueous solution of the dissolved monomer was heated to 40 °C. The intercalation-modified LMS was added, dispersed in the monomer-dissolved aqueous solution, and bathed for 10 min. Then, the temperature was raised to 65 °C. The initiator was added to the aqueous solution under nitrogen protection, and the reaction mixture was stirred for 5 h to obtain the polymerization product. The product was repeatedly rinsed with absolute alcohol and acetone, dried in an oven at 105 °C, and pulverized to obtain a white powder, which was organically modified inorganic composite, and the composite material was named LAZ. The preparation process is shown in Figure 1.

The composite LAZ was characterized by Fourier transform infrared spectroscopy (FT-IR) using Magna-IR 560 type from Nicolet (Nicoli Company, Ohio, OH, USA). The test method was potassium bromide compression with a 400–4000 cm^−1^ wavelength range and a resolution of 4 cm^−1^ [24]. The XRD analysis of the composite LAZ samples was examined on a D/MAX-RC type translational target X-ray diffractometer (Nippon Shinrikyo Co., Ltd., Kyoto, Japan). Test conditions were Cu target, K-rays, tube voltage 40 kV, current 100 mA, scanning speed 2 °/min, and composite LAZ sheet layer spacing calculated according to BragG′s formula, as given in the following equation:

(1) 2d⋅sinθ=λ

where *d* is the LMS sheet layer spacing; *θ* is the Bragg angle between incident light, reflected light, and reflected crystal surface; and *λ* is the incident light wavelength, taking the value 0.15406 nm [25].

TGA was performed using an analytical thermogravimetric instrument of Versa ThermHm TGA type (Thermo Fisher Scientific Inc., Waltham, MA, USA) for the characterization of the composite LAZ powder, with a measurement temperature range of 45–350 °C, a heating gradient of 5 °C/min, and a test atmosphere of high-purity argon [26].

LMS and the composite material LAZ were dispersed and dissolved in deionized water, frozen with liquid nitrogen, and vacuum-sprayed with metal. JSM-7800F (Nippon Shinrikyo Co., Ltd., Kyoto, Japan) was used to analyze the microstructures of LMS and the composite LAZ in water [27].

### 2.3. Performance Evaluation of Fluids

Rheological testing of composite LAZ solutions: The rheological properties of composite LAZ solutions were analyzed using an Anton Paar MCR102 rheometer (Anton Paar, Graz, Austria). The experiments were performed using a rotor model CC27, the measurement temperature was kept at 30 °C, and the changes in shear stress were tested as a function of the shear rate (shear rate ranging from 10 to 1000 s^−1^). The Herschel–Bulkley rheological model was used to describe the relationship between shear stress and shear rate of composite LAZ solutions [28].

The Herschel–Bulkley model, which is a combination of Bingham plastic and Power law models, was developed, as given by the following equation:

(2) $$\tau =\tau_{0}+K\gamma^{n}$$

where *τ* is the shear stress, Pa; *τ*_0_ is the yield stress, Pa; *K* is the consistency index, Pa·s^n^; *γ* is the shear rate, s^−1^; and *n* is the flow pattern index.

The viscoelastic index storage modulus G′ and loss modulus G″ were obtained using an MCR102 type rheometer in oscillatory mode with a fixed strain value for frequency scanning (strain *γ* = 1%; angular frequency ω = 0.1–10 rad/s) [29].

Test of the effect of the composite LAZ on the rheological properties of drilling fluids: The viscosity of drilling fluids was determined using a ZNN-D6 Six-speed viscometer (Qingdao Haitongda Special Purpose Instrument Company, Qingdao, China). American Petroleum Institute specifications were used as testing standards. Rheological properties such as AV, PV, and YP were calculated from readings at 600 rpm (*θ*_600_) and 300 rpm (*θ*_300_), as given by the following equations:

(3) $$AV=\frac{1}{2}\theta_{600}$$

(4) $$PV=\theta_{600}-\theta_{300}$$

(5) $$YP=0.511\left(2\theta_{300}-\theta_{600}\right)$$

In this study, hot rolling aging tests were conducted in an XGRL-5 rolling for high-temperature resistant properties. The composite LAZ was added into 400 mL 4% bentonite hydrated for 24 h, respectively. Prior to the measurements, the samples were stirred in a mixing cup under a high-speed homogenizer for 20 min and later transferred into stainless steel rollers and hot-rolled at different temperatures. The aging time was 16 h for all the samples. According to the comparison of the calculation results of Equations (3)–(5), the evaluation of temperature resistance was completed [21].

The composite LAZ was added into 400 mL brine drilling fluids of different concentrations. All samples were conducted in XGRL-5 rolling oven at different temperatures for 16 h. The rheological parameters before and after aging were measured using a six-speed viscometer and AV, PV, YP were calculated according to Equations (3)–(5); then, according to the comparison of data, the evaluation of salt resistance was completed [30].

## 3. Results and Discussion

### 3.1. Characterization

**Figure 2:**
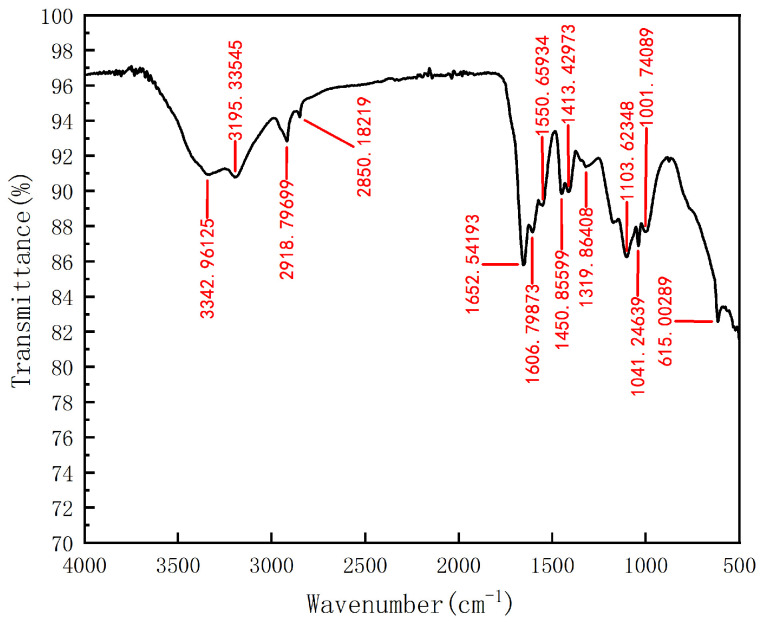
FT-IR spectra of the composite LAZ.

The infrared spectra of the composite LAZ are shown in Figure 2. The experimental results showed that a small absorption peak at 3342 cm^−1^ could belong to the telescopic vibrational absorption peak of -NH_2_ in AM. The stretching vibrational peak of the N-H bond is 3195 cm^−1^. Absorption peaks appeared at 2918 cm^−1^ and 2850 cm^−1^, which were the asymmetric and symmetric telescopic vibration absorption peaks of CH_2_, respectively, indicate the success of the organic modification. The cationic intercalating agent CTAB was inserted into the interlayer of LMS; 1652 cm^−1^ is the absorption peak for carbonyl. The C=O stretching vibrational absorption peak in the amide group is at 1606 cm^−1^. The characteristic absorption peaks for carboxylates are around 1550 cm^−1^ and 1450 cm^−1^. The absorption peaks appearing at 1413 cm^−1^, 1319 cm^−1^, and 615 cm^−1^ are characteristic LMS absorption peaks. The absorption peak at 1413 cm^−1^ is the bending vibrational absorption peak of the adsorbed water molecule –OH.

The absorption peak at 1319 cm^−1^ is the telescopic vibrational absorption peak of the Si-O bond in the lattice. The absorption peak at 615 cm^−1^ belongs to the bending vibration of Mg-OH-Mg; 1103 cm^−1^ and 1041 cm^−1^ are characteristic absorptions of the sulfonic acid group. The absorption peak at 1001 cm^−1^ is the telescopic vibrational absorption peak of -SO_3_^−^, indicating the successful introduction of AMPS into the polymer system. No characteristic absorption peaks for C=C were seen for the copolymer at 1620–1635 cm^−1^. The above peak data contain characteristic absorption peaks for the chain links of the LMS, AM, AA, and AMPS monomers, suggesting that the water-soluble monomers adequately carried out the polymerization reaction.

**Figure 3:**
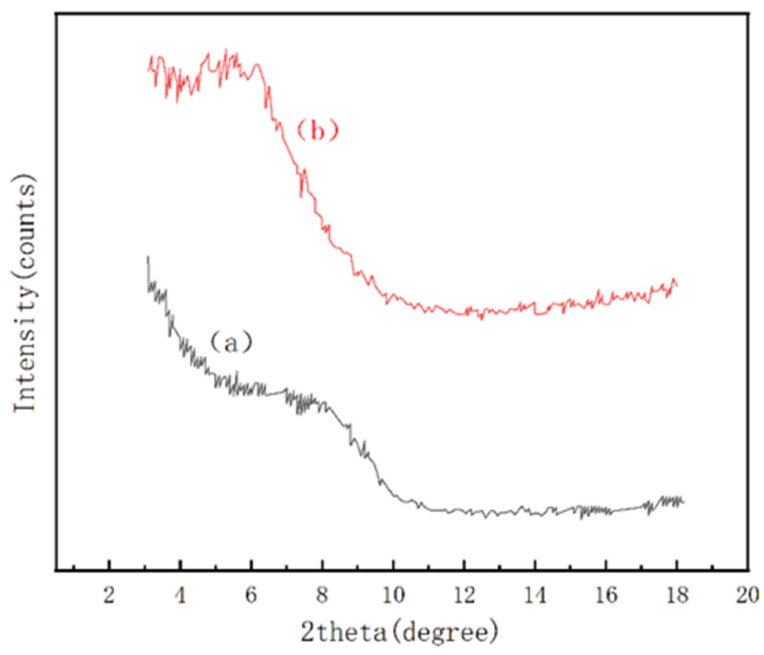
X-ray diffraction of different materials: (a) X-ray diffraction of LMS; (b): X-ray diffraction of composite LAZ.

The size of the sample interlayer spacing was determined by the position of the d_001_ diffraction peaks in the XRD pattern of the samples to determine the effect of modification of the composite LAZ. The XRD diffraction pattern of the composite LAZ is shown in Figure 3. The experimental results show that the d_001_ of unmodified LMS is 6.54°, its interlayer spacing is 1.35 nm, and the d_001_ of the composite LAZ is 5.18° with an interlayer spacing of 1.71 nm. The increase in the interlayer spacing indicates that the organic materials are arranged in a certain way in the interlayer of the sheet. At the same time, the peak shapes and the intensities of the diffraction peaks on the d_001_ side of the composite LAZ are also changed. The characteristic peaks of the composite LAZ are sharper and stronger than those without modifications.

**Figure 4:**
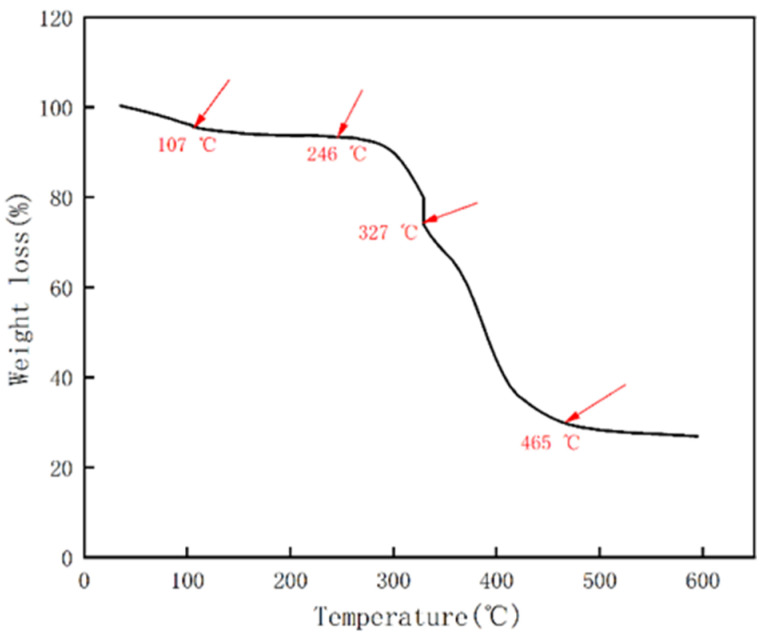
TGA curve of the composite LAZ.

The results of TGA performed on the composite LAZ are shown in Figure 4. The experimental results show that the weight loss of the composite LAZ can be roughly divided into three stages. The first stage is concentrated at 107–246 °C, which has a small mass of heat loss and a slow rate of weight loss, with a weight loss of 2.31%. The second stage occurs between 246 °C and 327 °C with a weight loss of 18.98%. The third stage occurs between 327 °C and 465 °C, where the weight loss is 44.25%. After 465 °C, the weight changes are small and maintained at approximately 27%.

**Figure 5:**
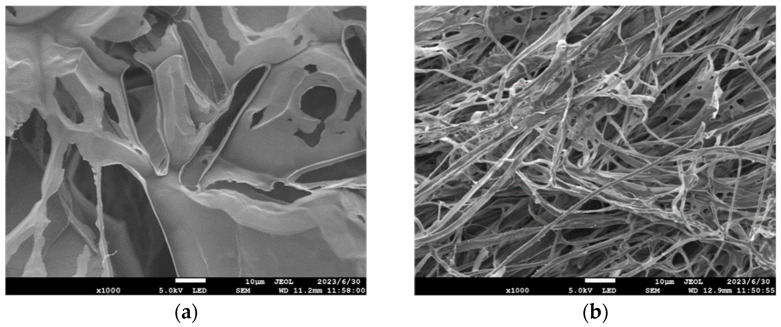
Microstructure images of different materials. (**a**) Microstructure of LMS; (**b**) microstructure of the composite LAZ.

The microstructure images of LMS solution and composite LAZ in an aqueous solution obtained using JSM-7800F field emission scanning electron microscope is shown in Figure 5. The experimental results of microstructure show that the LMS has a layered structure with a small number of holes similar to honeycomb. The composite material LAZ presents a complex reticulation structure. This reticulation structure intertwined in the aqueous solution stretch shows viscosity-enhancing properties conducive to enhancing the suspension performance of water-based drilling fluids.

### 3.2. Rheology of LAZ Solutions of Composites

**Figure 6:**
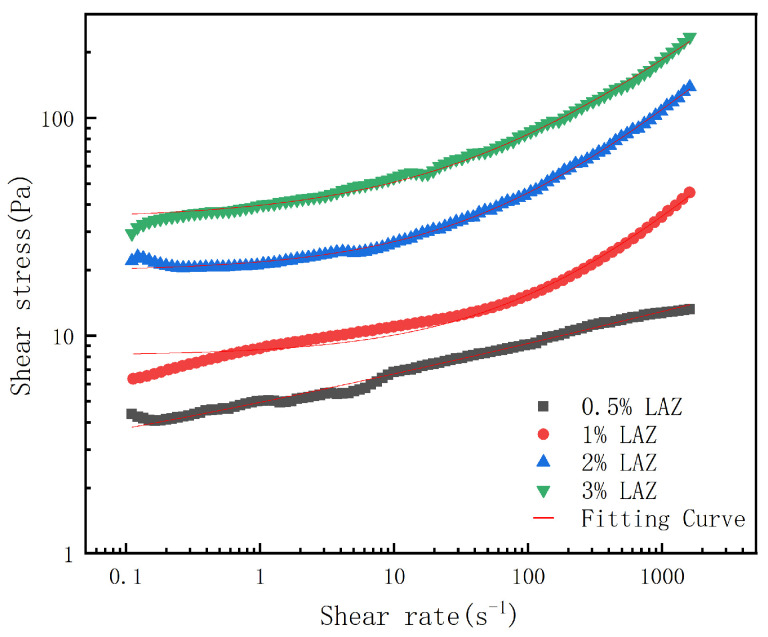
Shear rate–shear stress curves for composite LAZ aqueous solutions.

The composite LAZ has gel properties in an aqueous solution. The rates of disassembly and recovery of gel strength are important for shear thinning of drilling fluids at the wellbore and rock carrying in the annulus. The shear strain curves for aqueous solutions of different composite LAZ concentrations are shown in Figure 6. The corresponding parameter fitting results for the Herschel–Bulkley model are listed in Table 1. The experimental results showed that the aqueous solutions of LAZ with different concentrations of the composites had higher shear stresses with increasing shear rates, and the rheological data were fitted with high accuracy using the Herschel–Bulkley model. As the concentration of the composite LAZ in an aqueous solution increases, the yield stress value of the solution becomes stronger.

**Figure 7:**
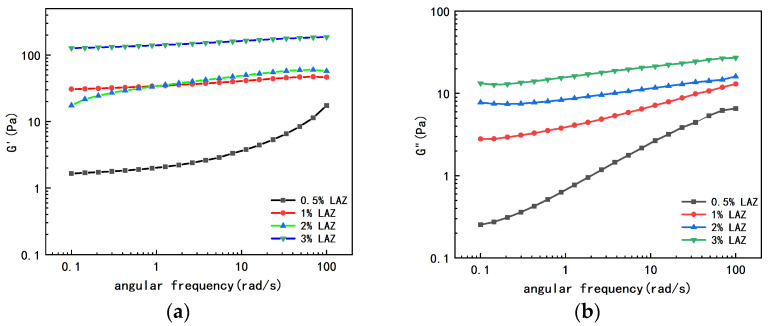
Viscoelasticity of the aqueous solutions of the composite LAZ with different concentrations. (**a**) Modulus of elasticity of composite LAZ; (**b**) viscous modulus of composite LAZ.

The viscoelasticity of the aqueous solutions of the composite LAZ at different concentrations was tested, and the results are shown in Figure 7. The experimental results show that the aqueous solution of the composite LAZ exhibits strong elastic characteristics with weak gel properties. The elastic modulus (G′) is much larger than the viscous modulus (G″). With the increase in the concentration of the composite LAZ in the aqueous solution, both the elastic modulus and the viscous modulus of the composite LAZ aqueous solution increase, indicating that the increase in the concentration of the composite LAZ is conducive to strengthening the strength of the aqueous solution.

### 3.3. Rheological Properties of the Composite LAZ as Viscosity Enhancer for Drilling Fluids

The 4% bentonite hydrated for 24 h was added to different concentrations of the composite LAZ. The drilling fluid was formed by high-speed mixing for 20 min to determine its rheological properties using a six-speed viscometer at 50 °C. The effect of the composite LAZ on the rheological properties of the drilling fluid is shown in Table 2. The experimental results showed that the drilling fluid’s viscosity and dynamic shear force increased significantly as the composite LAZ concentration increased from 0.1% to 1%. The increase in rheological parameters of the drilling fluid was small at 1–2% composite LAZ concentration. The best results were obtained by adding 1% LAZ to 4% bentonite-based slurry from drilling fluid, and the AV of the drilling fluid could be increased by a factor of 3.28 times compared to the 4% bentonite-based slurry.

The drilling fluid was formed by adding 4% bentonite hydrated for 24 h to 1% of the composite LAZ with high-speed mixing for 20 min and at hot rolling aging temperatures of 120 °C, 150 °C, and 180 °C for 16 h. The rheological properties of the drilling fluid before and after hot rolling aging were determined using a six-speed viscometer at 50 °C and compared with the unaged drilling fluid. The results of the experiments are shown in Table 3. The experimental results showed that the viscosity of the drilling fluids was decreased after hot rolling aging, which is due to the high-temperature effect causing part of the macromolecule degradation and chain breaking. Below the temperature conditions of 150 °C, the drilling fluid had good temperature resistance. The drilling fluid had better rheological properties. The rheological properties of the drilling fluid decreased greatly with hot rolling aging temperature at 180 °C. Experimental data indicated that the composite LAZ was greatly degraded under a temperature of 180 °C, and the viscosity-increasing performance of drilling fluid was reduced.

The composite LAZ was added to 4% bentonite hydrated for 24 h, and then 0 g, 4 g, 8 g, 12 g, and 20 g of sodium chloride was added after high-speed mixing for 20 min to form brine drilling fluids of different concentrations (percentage of sodium chloride mass to solvent water). A six-speed viscometer was used to determine the rheological properties of brine drilling fluid before and after the hot rolling aging of 150 °C for comparison. The results of these experiments are shown in Table 4. The experimental results showed that before hot rolling aging, the brine drilling fluid’s AV and YP showed a slow decreasing trend and then increased with an increase in the sodium chloride concentration.

After hot rolling aging at 150 °C, the AV of brine drilling fluid appeared to decrease and then increase, and the YP increased and then stabilized with the increase in the sodium chloride concentration. With the increase in the salt concentration, the composite LAZ and the hydrated electric double layer of bentonite were compressed, the repulsive force between the particles was weakened, the strength of the formed spatial network structure became smaller, and the YP and AV of brine drilling fluids decreased. The rheological properties before and after hot rolling aging at 150 °C showed that the brine drilling fluid had temperature and salt resistance, and the rheological properties of the brine drilling fluid changed with a small magnitude.

### 3.4. Tackiness Enhancement Mechanism Analyses

**Figure 8:**
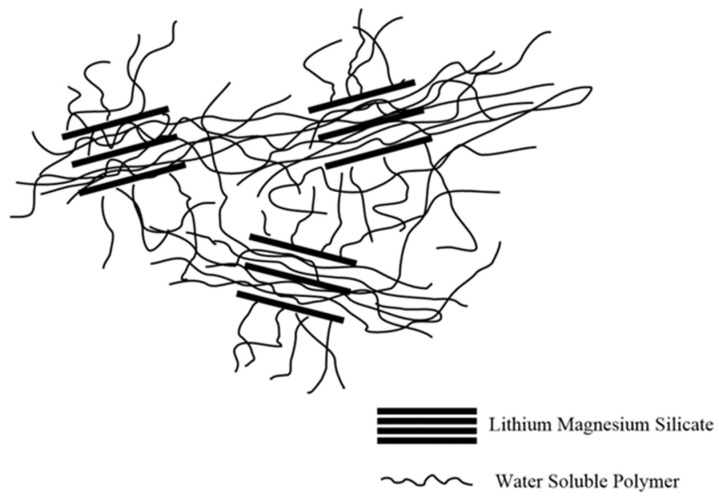
Schematic of the viscosity enhancement of the composite LAZ in water.

LMS is separated in the water to form multiple layers of individual flakes. The higher the dispersion degree, the better the viscosification effect. Based on the basic principle of intercalation polymerization, the composite LAZ may form a star-like supramolecular structure in an aqueous solution after combining the water-soluble polymer with LMS through intercalation polymerization. Star polymers possess at least three linear polymer arms that radiate from central cores. Their compact, three-dimensional globular geometries lead to uncommon chemical and physical properties, encompassing low viscosities and high densities of functional groups. Star-shaped molecules form more time-stable networks with larger and somewhat more distantly arranged aggregates compared to their linear counterparts [31,32,33]. The viscosity enhancement mechanism is shown in Figure 8. The presence of LMS, polymer chain charge, and mutual entanglement between polymers due to multiple forces play critical roles in the viscosity-increasing mechanism of the composite LAZ in an aqueous solution. Briefly, under the condition of multiple forces, the composite LAZ in the aqueous solution forms a complex network structure, thereby improving the viscosity of the aqueous solution.

## 4. Conclusions

In this paper, the composite, named LAZ, was prepared by combining the water-soluble monomers AM, AMPS, and AA with the intercalated LMS using intercalation polymerization. The basic chemical properties, rheological properties of the aqueous solution, and the effect of LAZ as a drilling fluid viscosity enhancer were studied. The following conclusions can be drawn based on this research:

- Based on the FTIR, XRD, TGA, and SEM characterization, we found that the modification of LMS was achieved via intercalation polymerization and that the interlaminar structures of LMS changed.
- The aqueous solution of the composite LAZ has shear dilution properties; as the shear rate increases, the shear stress becomes larger. The rheological properties of the composite LAZ are suitable for the Herschel–Bulkley model, and the yield stress value of the aqueous solution increases with the increase in the concentration of the composite LAZ. The elastic modulus (G′) is much larger than the viscous modulus (G″) and shows that the aqueous solution of the composite LAZ has weak gel properties.
- The composite LAZ can be used as a viscosity enhancer for drilling fluids. With the increase in the concentration of the composite LAZ, the viscosity and dynamic shear force of the drilling fluid increased significantly. The viscosity-increasing effect of adding 1% LAZ to 4% bentonite slurry was the most obvious, and the AV could be increased by 3.28 times. After hot rolling aging, the drilling fluid’s viscosity had a specific decrease. With the hot rolling aging temperature reaching 180 °C, the drilling fluid’s rheological properties decreased more, indicating that the composite LAZ polymer was degraded and that the chain was broken. The brine drilling fluid containing composite LAZ had temperature and salt resistance, and the change in rheological properties was small before and after the hot rolling aging temperature below 180 °C.

## 5. Future Perspectives

In this paper, the modified organic–inorganic composite LAZ was studied as a viscosity enhancer for water-based drilling fluid, which was beneficial for enhancing the rheological properties of drilling fluid. As a new material, the organic–inorganic composite material combines two or more compounds at the molecular level to make up for the shortcomings of the performance of a single material, synthesizes the advantages of multiple materials, and produces new properties, which can be applied in various fields. The application of such composite materials in other oilfield chemical treatment agents may improve the adaptability of these treatment agents in oilfields at high temperatures and in high salt environments.

## Figures and Tables

**Table 1:** Parameter fitting results of the LAZ Herschel–Bulkley model for composites.

Concentration (%)	*τ*_0_ (Pa)	*K* (Pa·s^n^)	n	R^2^
0.5	1.17	3.77	0.16	0.993
1.0	8.09	0.53	0.57	0.992
2.0	19.73	2.09	0.54	0.999
3.0	34.27	5.42	0.48	0.997

**Table 2:** Effect of the different concentrations of the composite LAZ on the rheology of drilling fluid.

Concentration (%)	*AV* (mPa·s)	*PV* (mPa·s)	*YP* (Pa)
0.0	12.5	4.0	8.69
0.1	15.0	6.0	9.20
0.3	17.5	7.0	10.73
0.5	22.0	9.0	13.29
1.0	41.0	20.0	21.46
2.0	40.0	18.0	22.48

**Table 3:** Effect of aging temperatures on the rheological properties of drilling fluids’ temperature (°C).

Temperature (°C)	*AV* (mPa·s)	*PV* (mPa·s)	*YP* (Pa)
Before aging	41.0	20.0	21.46
120	39.0	24.0	15.33
150	35.0	23.0	12.26
180	12.5	4.0	8.69

**Table 4:** Influence of rheology of saltwater drilling fluids.

Salt Concentration (%)	Test Condition	*AV* (mPa·s)	*PV* (mPa·s)	*YP* (Pa)	*YP/PV*
0.0	Before aging	41.0	20	21.46	1.07
	After aging	35.0	23	12.26	0.53
1.0	Before aging	38.0	19	19.42	1.02
	After aging	33.5	20	13.80	0.69
2.0	Before aging	34.0	16	18.40	1.15
	After aging	36.0	21	15.33	0.73
3.0	Before aging	46.0	23	23.51	1.02
	After aging	42.5	28	14.82	0.53
5.0	Before aging	43.0	21	22.48	1.07
	After aging	40.0	26	14.31	0.55

## Data Availability

Data are contained within the article.

## Abbreviations

CTAB	Cetyltrimethylammonium bromide
LMS	Lithium magnesium silicate
TEM	Transmission electron microscope
AMPS	2-acrylamido-2-methyl-propanesulfonic acid
MA	Maleic anhydride
AM	Acrylamide
AA	Acrylic acid
XRD	X-ray diffractometry
SEM	Scanning electron microscopy
FT-IR	Fourier transform infrared spectroscopy
TGA	Thermogravimetric analysis
